# Linker Design in Antibody-Drug Conjugates: Balancing Stability and Drug Release

**DOI:** 10.3390/pharmaceutics18080954

**Published:** 2026-08-03

**Authors:** Sara N. Albino, Margarida M. Domingos, Teresa R. Pacheco, Ana S. Carvalho, Miguel A. R. B. Castanho, Marco Cavaco

**Affiliations:** 1 Faculdade de Medicina, Universidade de Lisboa, Avenida Professor Egas Moniz, 1649-028 Lisboa, Portugal; sara.albino@edu.ulisboa.pt (S.N.A.); tr.pacheco@medicina.ulisboa.pt (T.R.P.); macastanho@medicina.ulisboa.pt (M.A.R.B.C.); 2 Faculdade de Farmácia, Universidade de Lisboa, Avenida Professor Gama Pinto, 1649-003 Lisboa, Portugal; 3 Fundação GIMM—Gulbenkian Institute of Molecular Medicine, Universidade de Lisboa, Avenida Professor Egas Moniz, 1649-028 Lisboa, Portugal; maria.domingos@gimm.pt; 4 Unidade Local de Saúde Santa Maria, Avenida Professor Egas Moniz, 1649-028 Lisboa, Portugal; 5 Center for Mathematical Studies, Faculdade de Ciências, Universidade de Lisboa, C6—Piso 1, Sala 6.1.03, 1749-016 Lisboa, Portugal; asocarvalho@ciencias.ulisboa.pt

**Keywords:** antibody–drug conjugates, peptide linker design, tumour-associated enzymes, therapeutic index, oncology

## Abstract

Antibody–drug conjugates (ADCs) have emerged as a powerful class of targeted therapeutics in many clinical areas, such as in oncology. Despite their efficacy, the onset of adverse events has been a major drawback in their clinical use. Among other explanations, the clinical performance of the ADCs has been associated with the chemistry of the linker connecting the antibody and payload. Linkers determine plasma stability, intracellular activation, and payload diffusibility, thereby influencing the therapeutic index, off-tumour toxicity, and by-stander activity. Mechanistic insights increasingly show that linker–payload properties govern catabolite permeability and intratumoral distribution, particularly in antigen-heterogeneous settings. Current developments include enzyme-cleavable and tumour-selective linkers, polarity-modulating masking strategies, alternative self-immolative spacers, and dual-trigger systems designed to enhance selectivity and decouple efficacy from toxicity. In parallel, linker behaviour intersects with broader mechanisms of tumour resistance. This review focuses on understanding these processes, which are essential for designing the next generation of linkers capable of improving stability, safety, and long-term therapeutic effectiveness across diverse tumour contexts.

## 1. Introduction

Cancer remains among one of the leading causes of morbidity and mortality worldwide, with the latest GLOBOCAN 2022 estimates indicating approximately 20 million new cases and 9.7 million cancer-related deaths in a single year [1,2]. Despite advances in cancer therapeutics, the effective delivery of highly potent cytotoxic agents continues to be limited by insufficient tumour selectivity and dose-limiting toxicities. This highlights a central challenge in oncology: how to maximize tumour cell killing while minimizing systemic damage.

Conventional systemic chemotherapy has long been the most common therapeutic option for cancer treatment. However, owing to its lack of specificity towards tumour cells/tissues, it is associated with significant adverse effects on healthy tissues [3]. Dose intensity is often constrained by bone marrow suppression, gastrointestinal and neurological toxicity, and cumulative organ damage, which will limit long-term disease control despite potent antitumour activity. These constraints have driven efforts to develop therapeutics that retain the cytotoxic activity of chemotherapy while improving selective delivery to tumour cells [3,4,5].

**Figure 1:**
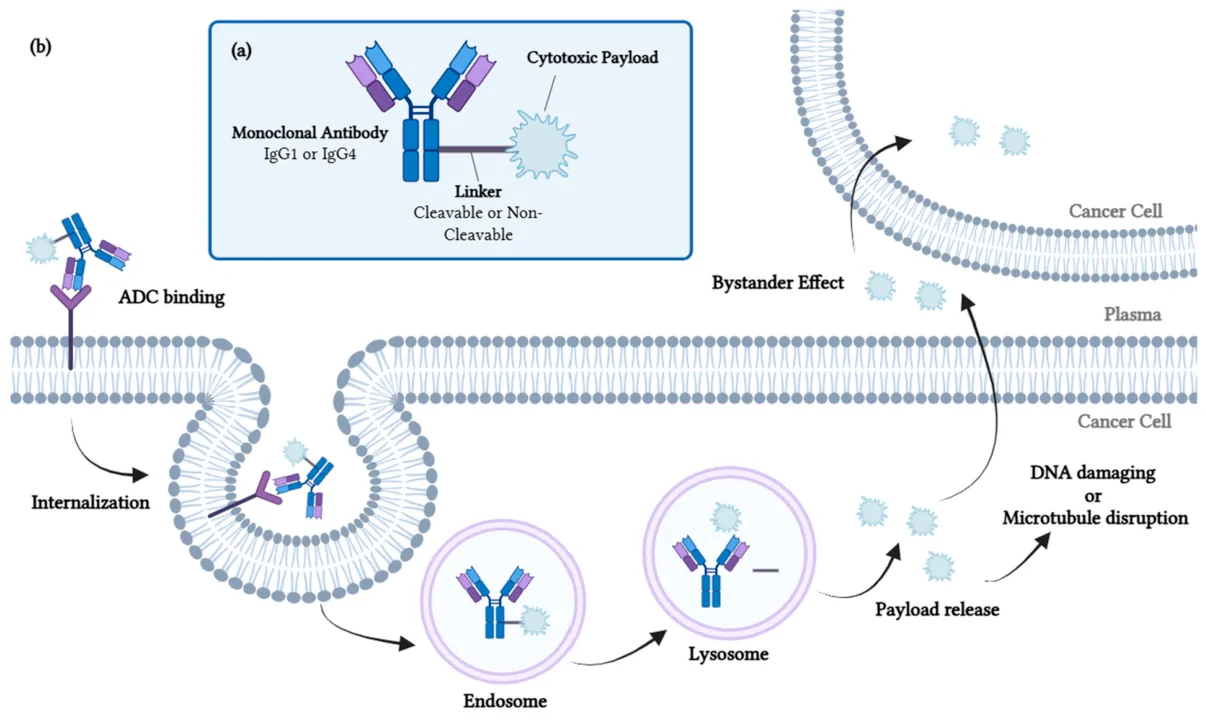
Structure and Mechanism of Action of ADCs. (**a**) ADC combines three key components: a monoclonal antibody that binds the antigen, a cytotoxic payload that targets specific components and a covalent linker that connects the antibody and the payload. (**b**) ADC mechanism of action, including the key sequential steps.

Antibody–drug conjugates (ADCs) have appeared as a promising solution to address these limitations through the conceptualization of chemotherapy as a targeted biologic-small-molecule hybrid (Figure 1). ADCs are modular constructs composed of 3 key elements: a targeting monoclonal antibody (mAb), cytotoxic payloads, and a chemical linker. While the antibody assures selectivity by recognizing a tumour-associated antigen, decreasing off-target effects, and the payload provides potent cell-killing activity, allowing efficient cancer cell elimination, the linker governs the spatial and temporal control of drug release [3,6,7,8]. By delivering the highly cytotoxic drugs directly to cancer cells while minimizing the exposure of normal tissues, ADCs are intended to improve the therapeutic index relative to traditional systemic chemotherapy [3,8,9]. Over the past decade, this strategy has been increasingly validated in the clinic, with a growing number of approvals across hematologic malignancies and solid tumours, establishing ADCs as a major pillar for modern oncology [3,10]. Several ADCs have been approved by the Food and Drug Administration (FDA) and the European Medicines Agency (EMA) [11,12] (Table 1), and many others in Phase III clinical trials (Table 2).

**Figure 2:**
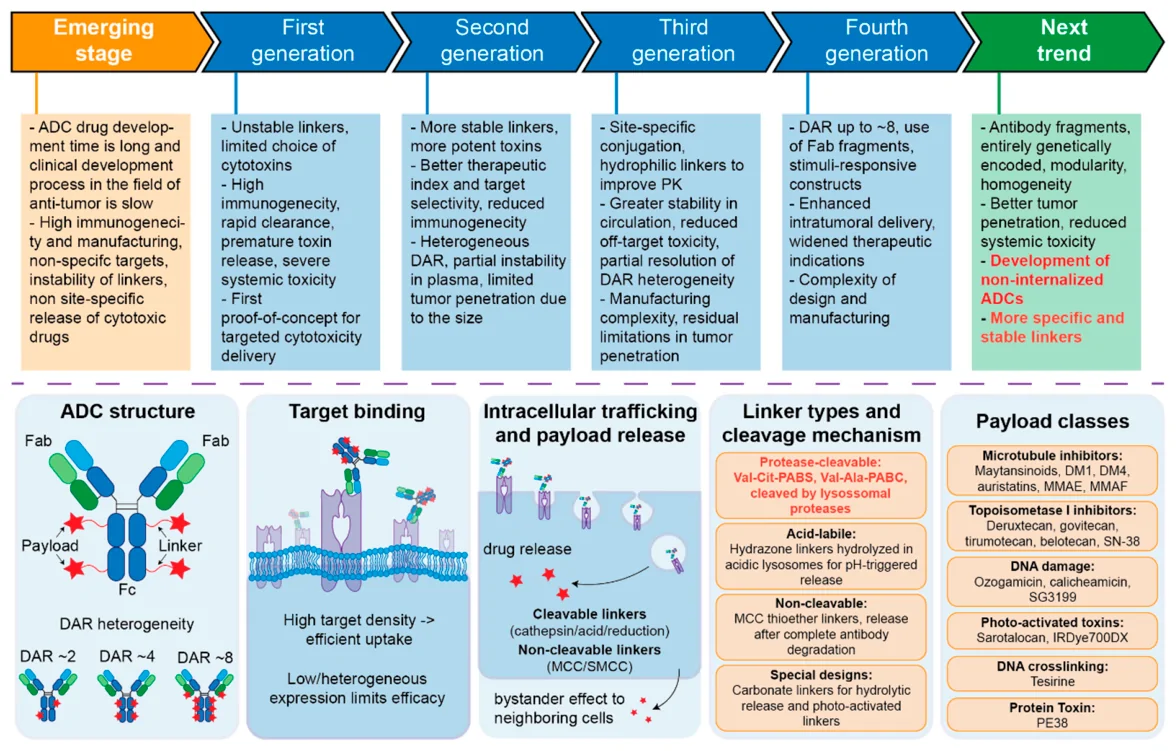
The development of antibody-drug conjugates (ADCs). (**Top**) The development stages of ADCs and the next. (**Bottom**) Key characteristics of approved ADCs. DAR, drug-to-antibody ratio; PK, Pharmacokinetic; Fc, Fragment crystallizable; Fab, Fragment antigen-binding.

The clinical development of ADCs has revealed design challenges, particularly regarding linker stability and drug release (Figure 2). First-generation ADCs, such as Gemtuzumab ozogamicin, relied on acid-labile hydrazone linkers and reducible disulfides, which were prone to premature cleavage in circulation or mildly acidic normal tissues. This instability led to inadequate tumour selectivity, significant off-target toxicity, and a narrow therapeutic window [6,13]. These early limitations established a fundamental principle in ADC design: an optimal balance between systemic stability must be achieved, so that the intact conjugate reaches the tumour, and efficient intracellular drug release occurs [3,6,13]. Subsequent generations of ADCs have addressed these challenges through improved linker chemistries. Second-generation ADCs introduced more stable non-cleavable thioether linkers that minimized premature drug release while still enabling effective payload release after lysosomal degradation of the antibody. More recently, third-generation ADCs combined optimized antibody engineering, highly potent next-generation payloads, and carefully tuned cleavable linkers that remain stable in circulation, but are efficiently activated within tumour cells, leading to improved clinical performance and expanded therapeutic indications [3,6,14].

These advances collectively emphasize the central role that linkers play in determining ADC behaviour in vivo. Rather than acting as a passive connector, the linker is now recognized as a critical design element that influences multiple aspects. Linker chemistry works as a primary design force that needs to be carefully optimized with antibody engineering, payload selection, and conjugation technology to obtain robust therapeutic indices [13,15]. The role of the linker extends across all critical aspects of ADC function: it determines systemic stability and the risk of premature drug release, thereby influencing systemic toxicity and therapeutic index. It also affects internalization and intracellular processing, because of its influence on how ADC is trafficked and processed within endosomes and lysosomes, where many cleavable linkers are activated [16,17]. Linkers also modulate the bystander effect, and hence the extent to which surrounding antigen-low tumour cells and normal cells are exposed to the drug, through controlling the form, charge, and diffusibility of the released payload. Finally, linker structure contributes to overall hydrophobicity and aggregation propensity, shaping tissue distribution, off-target uptake, and safety, and it also interacts with conjugation strategies to influence drug-to-antibody ratio (DAR) and product heterogeneity [3,6,13,18,19].

Taken together, these considerations highlight the central role of linker chemistry in shaping ADC behaviour, stability, and therapeutic index. This review focuses on enzyme-cleavable and tumour-selective linker strategies, and examines their biological rationale, design principles, and translational implications for modern ADC development.

## 2. ADC Architecture and the Central Role of the Linker

### 2.1. Core Components of ADCs

ADCs are modular biologics composed of three main components, each contributing distinct functional properties to the final therapeutic agent [8] (Figure 1). The mAb, usually a human or humanized IgG1 or IgG4, defines target selectivity and provides a pharmacokinetic (PK) framework. The payload, a small-molecule drug, is typically selected from well-characterized chemotype classes, such as microtubule-disrupting agents, topoisomerase inhibitors, or DNA-alkylating agents, engineered to exhibit sub-nanomolar potency against tumour cells while maintaining suitable physical and chemical properties compatible with conjugation [3,8,13,20]. Finally, the linker, which constitutes the chemical bond connecting the mAb and payload [6,13].

The linker composition and reactivity directly impact how and where the payload is released, with structural features determining plasma stability and susceptibility to specific cleavage mechanisms [6,18]. Linkers are usually short (often 5–20 atoms) and can range from simple aliphatic chains to complex synthetic scaffolds with enzymatic labile motifs, hydrophilic spacers and self-immolative trigger elements. Critically, linkers need to be compatible with the chemical functional groups on the mAb but not significantly impair antibody binding or biophysical properties. They must also endure both the manufacturing process and systemic circulation long enough to allow efficient tumour uptake without releasing the payload prematurely into the bloodstream [6,17,18].

Linker’s structure affects almost every stage of ADC disposition and function. It affects the spatial relationship between mAb and payload, altering tumour penetration and intracellular trafficking. For example, early DM1-ADCs incorporating cyclohexyl-based SMCC linker were characterized by the hydrophobic nature of the resulting linker–payload construct, resulting in increased aggregation and impaired intracellular trafficking, leading to accelerated hepatic clearance. In contrast, more flexible linkers such as SPP or sulfo-SMCC improved solubility and internalization efficiency. Biophysical properties, such as hydrophobicity, length, and rigidity of the linker scaffold, contribute to the overall biophysical profile of the conjugate, affecting clearance kinetics, tissue distribution, and the propensity to form high-molecular-weight aggregates [3,6,13]. The use of hydrophobic linkers used in MMAE-ADCs increased aggregation and liver uptake, prompting the development of PEGylated linkers. It determines the route and kinetics of payload release in different cellular compartments, and it can alter the presentation of antibody epitopes, Fc domain interactions, and off-target pharmacology [3,6,17,20]. Therefore, linker engineering appears to be a great opportunity to fine-tune ADC developability and the therapeutic index, independent of target selection and payload optimization.

### 2.2. Drug-to-Antibody Ratio, Conjugation Strategy, and Heterogeneity

The DAR is a quantitative descriptor of ADC composition defined as the average number of drug molecules covalently attached to each mAb molecule. Traditional conjugation methods targeting multiple lysine residues or interchain disulfide cysteines typically yield heterogeneous mixtures comprising species with DAR values from 0 unconjugated antibodies to 8 or higher heavily loaded molecules, with each subpopulation presenting distinct stability, clearance, and potency [3,19,21]. A well-established example is Trastuzumab-DM1, whose lysine-based conjugation produces a broad DAR 0–8 distribution, with each subpopulation exhibiting distinct stability, clearance, and potency. Although high-DAR species can deliver more payload, they often exhibit increased hydrophobicity, greater aggregation propensity, faster plasma clearance, and higher off-target uptake. Conversely, low-DAR species often exhibit excellent plasma stability and prolonged half-lives but may have insufficient payload to kill all contacted tumour cells [3,19]. This heterogeneous distribution of DAR within a population can result in a “mixed population” whose average behaviour may not accurately reflect the properties of any single species and complicate the interpretation of results [19,21].

Recent studies highlight that linker chemistry and conjugation strategies are inextricably connected, due to both the number and position of conjugation sites, combined with the physicochemical properties of the linker itself, determining the potential DAR distribution and the overall biophysical characteristics of the ADC [15,21]. In the case of Brentuximab vedotin, the hydrophobic MMAE payload combined with the linker limits manufacturable DAR to approximately 4, as higher-DAR species aggregate and are cleared rapidly. Conversely, hydrophilic linkers can support higher DAR values: Trastuzumab-deruxtecan achieves a DAR of 8 due to its highly hydrophilic tetrapeptide-based linker, which offsets payload hydrophobicity and prevents aggregation. Preclinical studies using polysaccharide or PEG-based linkers further demonstrated that hydrophilic scaffolds can sustain DAR values of 6–10 without compromising their solubility or stability.

Site-specific conjugation methods, such as engineered cysteines (THIOMABs), glycan remodelling, enzymatic tags or biorthogonal click handles, enable the production of ADCs with defined DAR values, usually 2 or 4, and fixed conjugation sites, yielding more homogeneous products with improved PK predictability [3,7,15,21]. In these strategies, linker design needs to be tuned to offset any hydrophobic burden introduced by diverse payloads because hydrophobic linkers can impair the benefits of site-specific attachment through promoting aggregation and rapid clearance, whereas hydrophilic linkers can enable site-specific ADCs with higher DAR without compromising solubility or stability [3,6,15]. Emerging computational approaches further underscore that DAR, hydrophobicity and linker structure are tightly coupled design dimensions that can be optimized together [22].

### 2.3. Classification and Mechanistic Roles of Linkers

**Figure 3:**
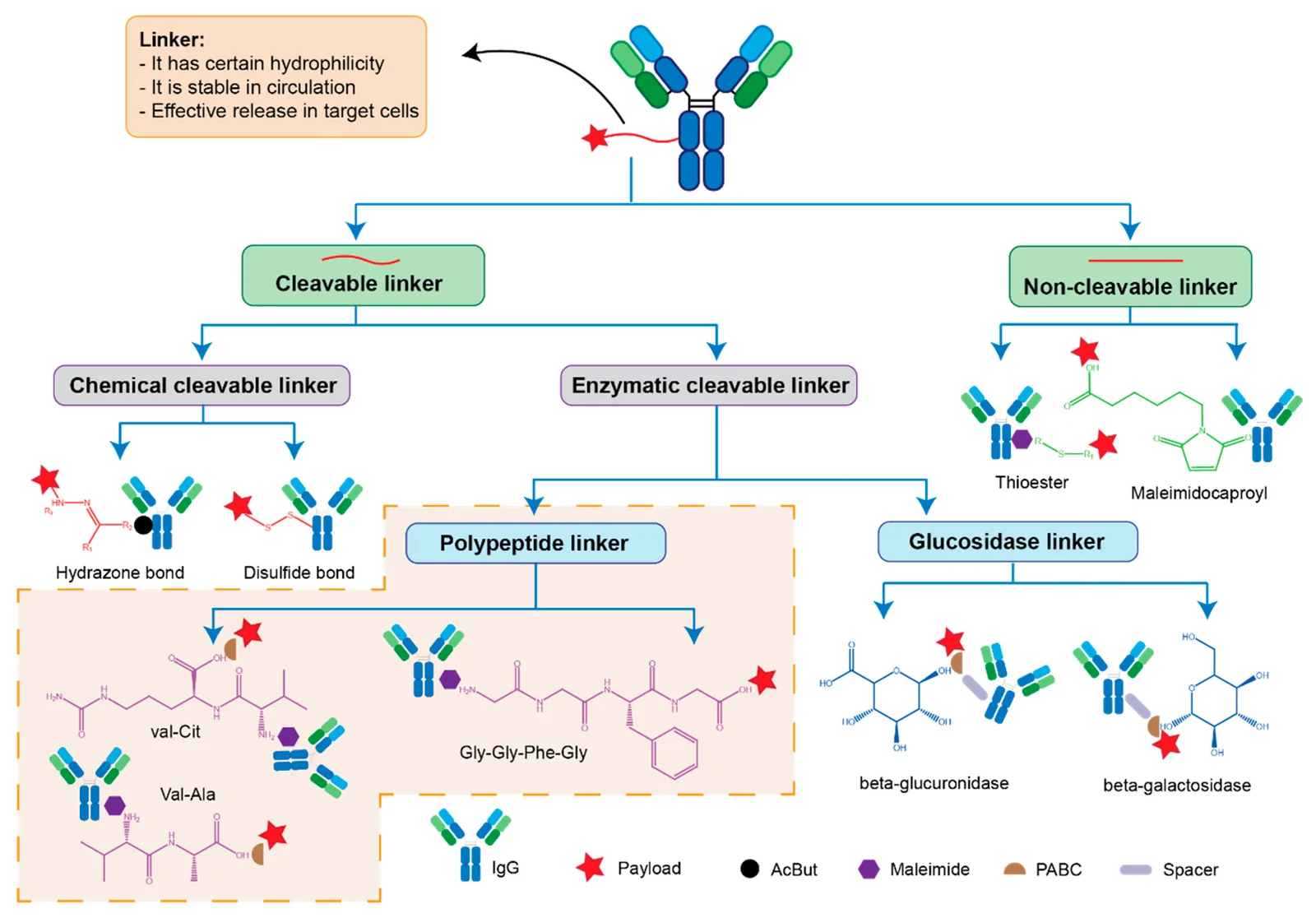
Classification of linkers in the antibody-drug conjugates (ADCs). Linkers of ADCs are classified into two categories: cleavable and non-cleavable linkers. Cleavable linkers consist of seven subtypes, which can be further divided into chemical cleavable and enzymatic cleavable linker.

From a mechanistic perspective, linkers are usually classified into two main categories, non-cleavable and cleavable (Figure 3), with distinct implications for where and how the payload becomes active [6,13,15].

Non-cleavable linkers are designed to remain intact throughout systemic circulation and extracellular processing, with payload release occurring only after the internalized ADC undergoes complete proteolytic degradation of the mAb scaffold in the lysosomal compartment [6,13]. The payload release occurs after lysosomal proteolysis of the mAb scaffolds, generating a charged drug-amino acid catabolite, which potentially impairs membrane permeability and limits bystander effects, and the diffusion of active drug from target cells to neighbouring tumour cells [13,15]. This design offers plasma stability and chemical robustness, and relies on lysosomal degradation as a natural, selective activation mechanism [13].

In contrast, cleavable linkers are engineered to undergo specific chemical or enzymatic scission under defined intracellular or tumour microenvironment (TME) conditions, such as proteolytic activity, low pH, or redox changes, releasing a more hydrophobic, membrane-permeable drug capable of efficiently diffusing and killing neighbouring cells [6,13,15,16]. Subtypes include:

- pH-sensitive linkers, like hydrazones or acylhydrazones, are stable at neutral pH but are efficiently hydrolysed in the acidic environment of endosomes and lysosomes, where they release free drug for diffusion through lysosomal membranes. These types of linkers were prominent in early-generation ADCs but are now less commonly used because of problems related to premature release in mildly acidic tissues and unpredictable stability throughout different pH ranges [13,15].
- Redox-responsive linkers usually incorporate disulfide bonds that are reduced in the cytoplasm through glutathione or protein disulfide isomerases. However, they remain intact in oxidative extracellular and plasma environments. Although used in some clinical mAbs, concerns about non-selective reduction in normal tissues with glutathione (GSH) levels have reduced their prominence [6,13].
- Enzyme-cleavable linkers, usually peptide-based motifs, such as Val-Cit or GGFG, that are recognized and hydrolysed by lysosomal cathepsin proteases, cathepsin B, L, and S, following ADC internalization. These linkers exploit the high activity of cysteine proteases in the lysosomal compartment of tumour cells while remaining relatively stable in plasma and normal tissues. Other alternatives for enzyme-responsive linkers include those with glycosidic motifs that are cleaved by β-galactosidase or are designed around matrix metalloproteinase (MMP)-sensitive sequences active in the tumour microenvironment, presenting alternative or complementary enzymatic triggers [3,6,23,24].

Among the cleavable designs, enzyme-cleavable peptide linkers have become the main focus of drug research and development, as they offer an attractive combination of high plasma stability, due to lysosomal cathepsins having low or absent activity in extracellular fluids, efficient intracellular cleavage, and the potential for further optimization toward enhanced tumour selectivity through substrate design and protease profiling [6,15,23]. Mechanistic and structural data showed that amino acid identity and linker length can modulate cathepsin specificity and cleavage kinetics, providing a roadmap for rational design of second- and third-generation peptide linkers with improved selectivity for tumour-associated proteases and reduced off-target activation [6,23]. Consequently, linker choice is not a local chemical detail but a central design decision that must be optimized alongside antibody architecture, antigen biology, internalization kinetics, payload properties, and conjugation method to achieve clinically meaningful improvements in efficacy and safety [6,13,15,25].

## 3. Tumour-Associated Enzymes as Triggers for Linker Cleavage

Compared to normal tissues, many solid and hematologic tumours exhibit remodelling of their proteolytic and lysosomal machinery, with several enzyme families consistently overexpressed or hyperactivated (Table 3). These enzymes concentrate in enlarged lysosomes, invadopodium and TME, where they contribute to extracellular matrix (ECM) degradation, growth factor activation, invasion, metastasis, angiogenesis and therapy resistance. Crucially, this results in higher and spatially focused enzymatic activity in tumours compared with healthy tissues, creating biochemical niches that differ sharply from the systemic circulation. This dysregulated activity can also be exploited as a biochemical trigger, making tumour-associated enzymes highly attractive as selectivity filters for ADC linkers [24,26,27,28,29].

### 3.1. Lysosomal Proteases

Lysosomes are central hubs for protein turnover and signalling, and many solid and hematologic tumours exhibit increased lysosomal biogenesis and protease content. Lysosomal proteases are found in tumour cell lysosomes and, sometimes, in the pericellular space, and their activity is significantly elevated in highly metastatic tumour variants, such as the B16F10 melanoma model or aggressive mammary carcinoma models, compared to the less aggressive ones [30,31]. Recently, this observation was extended to multiple cancer types, in which upregulated lysosomal proteases facilitate degradation of the extracellular matrix, activation of growth factors, and modulation of cell death pathways [26,29].

Due to internalized ADCs being trafficked through endosomes to lysosomes, this compartmental enrichment of proteolytic activity has been directly exploited in lysosomal-cleavable peptide linkers. Linkers with motifs that are recognized by lysosomal proteases are designed to remain intact in plasma and extracellular fluids but be efficiently hydrolysed in the acidic, enzyme-rich lysosomal lumen, triggering self-immolation and payload release [16,32,33]. The elevated lysosomal protease activity in tumours compared with normal tissues promotes a functional selectivity window for linker design, although inter-tumoral heterogeneity and species differences must be considered [16].

### 3.2. Cathepsins

Cathepsins are a subfamily of lysosomal proteases, including cysteine cathepsins, aspartic cathepsins, and serine cathepsins, that are central to protein turnover and antigen processing. Among them, cathepsin B has received particular attention in oncology because its expression and activity are elevated in a wide range of tumours, including breast, pancreatic, colorectal, prostate, and melanoma [26,30,34,35].

**Table 3:** Overexpressed enzymes in the tumor microenvironment.

Family	Enzyme	Location	Substrate	Tumor	Function	Pathophysiological Role	Prognosis	References
Cathepsin (Cysteine)	B	Pericellular space; lysosomes	Broad spectrum	Various	Degradation of ECM components, activation of other proteases and processing of growth factors and cytokines	General protein turnover in lysosomes, antigen processing, regulation of apoptosis, and autophagy	Increased invasion, metastasis, and poor prognosis in several cancers	[36,37]
	L	Lysosomes	Broad spectrum	BC, HNC, LC	Degrades ECM proteins and basement membrane components, contributes to EM, invasion and metastasis	Protein turnover, MHCII antigen processing, regulation of cell cycle, and apoptosis	Advanced disease and worse outcome in cancers	[26,37,38]
	S	Lysosomes and endosomes	ECM components	Solid tumors	Degrades ECM	Critical for MHCII invariant chain degradation and antigen presenting	More aggressive disease and poor survival in some cohorts	[26]
	K	Lysosomes	Collagen	mBone, BC, PC, melanoma	Degrades type I collagens and bone matrix	Potent collagenase	Bone invasion and metastasis	[34]
Cathepsin (Aspartic)	D	Lysosomes	Many proteins and pro-hormones	BC, CRC, OC	Degrades protein in lysosomes at an acidic pH; activates precursors of biologically active proteins in pre-lysosomal compartments or specific cells	Pro-enzyme and mature forms both biologically active	Emulates cancer cells’ proliferation, fibroblast outgrowth, angiogenesis, and metastasis	[29,36,37]
Cathepsin (Serine)	G	Azurophilic granules of neutrophils	ECM components	Carcinomas	Degrades ECM components, activates other proteases	Modulates inflammation	Pro-inflammatory, pro-TME	[39]
MMP	2	ECM, basement membranes and cell surfaces	Type IV collagen and gelatine	BC, CRC, LC, GC, melanoma	Degrades substrates in basement membranes; releases ECM-bound growth factors	Promotes invasion and angiogenesis	Lymph node metastasis, advanced stage and poor survival	[40,41,42]
	9	ECM, basement membranes and cell surfaces	Type IV collagen and gelatine	BC, CRC, LC, GC, melanoma	Degrades substrates in basement membranes; releases ECM-bound growth factors	Promotes invasion and angiogenesis	Lymph node metastasis, advanced stage and poor survival	[40,41,42]
	11	ECM	Collagen and ECM components	Carcinomas	Degrades proteoglycans, laminin and fibronectin	Promotes invasion and angiogenesis	Metastasis and poor outcome	[27]
	13	ECM	Collagen and ECM components	Carcinomas	Degrades collagens in the ECM	Promotes invasion and angiogenesis	Metastasis and poor outcome	[27,41]
	14	Cell surfaces	Pro-MMP2 and collagen	BC, melanoma	Activates pro-MMP2 and degrades collagen	Pericellular proteolysis and cancer cell invasion	Invasion and poor prognosis	[27,41]
ADAM	10	Cell surfaces	Cell-surface proteins	BC, CRC, GC	Enhances the shedding of inflammatory mediators, adhesion molecules, and growth factor ligands	Growth factor signaling and immune modulation	Aggressive behavior and poor prognosis	[43,44,45]
	17	Cell surfaces	Cell-surface proteins	BC, CRC, GC	Enhances the shedding of inflammatory mediators, adhesion molecules, and growth factor ligands	Growth factor signaling and immune modulation	Aggressive behavior and poor prognosis	[43,44,45]
Glycosidase	β-glucuronidase	Lysosomes	β -D-glucuronic acid residues	BC, CRC, CC, pancreatic	Hydrolyses β-D-glucuronic acid residues from glycosaminoglycans and glucuronide conjugates	Deconjugats glucuronic acid	Advanced stage and worse prognosis	[46,47]
	β-galactosidase	Lysosomes	β -D-galactose	Liver, OC, PC, CRC, BC, gliomas	Removes β-D-galactose from glycoconjugates	Glycan turnover	Not well defined	[48,49]
Protease (Serine)	Matriptase	Cell surfaces	Zymogens and ECM components	BC, CRC, Pancreatic	Activates pro-uPA, pro-MMPs and other zymogens; degrades ECM components	Promotes invasion and EMT	Increased invasion, metastasis and poor survival in several cancers	[50,51,52]
	TMPRSS3	Cell surfaces	Zymogens and ECM components	BC, CRC, Pancreatic	Activates pro-uPA, pro-MMPs and other zymogens; degrades ECM components	Promotes invasion and EMT	Increased invasion, metastasis and poor survival in several cancers	[50]
	TMPRSS4	Cell surfaces	uPA	Pancreatic, thyroid, CRC	Activates pro-uPA	Promotes invasion	Increased invasion, metastasis and poor survival in several cancers	[51,52,53]
	TMPRSS13	Cell surfaces	Zymogens and ECM components	BC, CRC, Pancreatic	Activates pro-uPA, pro-MMPs and other zymogens; degrades ECM components	Promotes invasion and EMT	Increased invasion, metastasis and poor survival in several cancers	[50,51,52]
	uPA	Cell surfaces	Zymogens and ECM components	Various	Activates pro-uPA, pro-MMPs and other zymogens; degrades ECM components	Promotes invasion and EMT	Increased invasion, metastasis and poor survival in several cancers	[50,51,52]
Specific Protease (Ubiquitin)	USP4	Nuclear or cytoplasmic	Ubiquitin	BC	Important determinant for the crosstalk between the TGF-β and AKT signalling pathways	Regulates oncogenic pathways	Link to proliferation, therapy resistance and poor prognosis	[54,55,56]
	USP39	Nuclear or cytoplasmic	Ubiquitin	BC	Removes ubiquitin from target proteins; involved in mRNA splicing	Tumor suppressor	Link to proliferation, therapy resistance and poor prognosis	[55,56,57]

BC, breast cancer; CC, cervical cancer; CRC, colorectal cancer; ECM, extracellular matrix; EMT, epithelial-to-mesenchymal transition; GC, gastric cancer; HNC, head and neck cancer; LC, lung cancer; MHC, major histocompatibility complex; MMP, metalloproteinase; OC, ovarian cancer; PC, prostate cancer; TME, tumor microenvironment.

These enzymes are associated with invasive and metastatic phenotypes, contributing to ECM remodelling, epithelial–mesenchymal transition (EMT), and modulation of apoptosis and autophagy pathways. The expression and activity of these enzymes show “patchiness” expression and activity within tumours, with localized hotspots that may drive focal invasion and metastasis [26,31,58].

From a drug delivery perspective, cathepsins are a primary enzymatic trigger exploited by cathepsin-cleavable ADC linkers. Peptide motifs, like Val-Cit and GGFG, have been shown to be efficiently cleaved by lysosomal cathepsins, and many approved ADCs, such as Brentuximab-vedotin, Polatuzumab-vedotin, Enfortumab-vedotin and Trastuzumab-deruxtecan, rely on these reactions to release their payloads after internalization [32,33]. Medicinal-chemistry design, assisted with protease substrate profiling, demonstrates that a careful selection of P1-P4 residues can bias cleavage toward specific cathepsins and tune stability versus activation across species [16,26,33].

However, cathepsins are not tumour exclusive. They are active in normal lysosomes and can be secreted into the extracellular space under inflammatory conditions [59]. Therefore, tumour selectivity arises from differences in activity level, localization, and trafficking, rather than expression alone [33,59].

### 3.3. MMPs in the Tumour Microenvironment

MMPs are zinc-dependent endopeptidases that play major roles in ECM remodelling, angiogenesis, and immune cell trafficking. The expression of MMP-2, MMP-9, and MMP-14 is frequently upregulated in tumour cells, stromal fibroblasts, and infiltrating immune cells, particularly in breast cancer, glioblastoma, gastric cancer, and melanoma. Their proteolytic activity enables cancer cells to breach basement membranes, degrade interstitial collagens, shed adhesion molecules, and release ECM-bound growth factors and cytokines, thereby promoting invasion and metastatic dissemination [27,60].

MMP activity is often concentrated at the invasive front of tumours and in perivascular niches, where localized ECM degradation correlates with increased metastatic potential. This spatially and tumour-biased activity overexpression motivated extensive exploration of MMP-cleavable peptides as triggers for prodrugs and nanocarriers, with cleavage in the TME unmasking or releasing active drugs near tumour cells [43,60].

MMP-sensitive motifs have been successfully deployed in peptide-drug conjugates and polymeric drug delivery systems (DDSs), highlighting that MMP-rich microenvironments can be harnessed for tumour-localized drug activation. However, since some MMPs are also upregulated in inflammatory and remodelling processes, it is required to have a careful profiling of MMP expression and activity in tumour versus normal tissues is required to avoid off-tumour activation [61,62].

### 3.4. Glycosidases

Glycosidases are lysosomal exoglycosidases involved in the degradation of glycosaminoglycans and glycoproteins. In several types of tumours, including breast, prostate, lung, and colorectal cancers, b-glucuronidase, and b-galactosidase, are found at elevated levels in tumour tissues and associated extracellular fluids, especially in necrotic regions where lysosomal contents are released due to cell death [28,48,49].

This biology has been exploited in glycosidase-responsive prodrugs, where the active drug is masked with a sugar moiety and remains inactive until the tumour milieu is enzymatically cleaved. B-Glucuronide prodrugs of doxorubicine and camptothecin, for example, showed enhanced tumour selectivity in xenograft models with high b-glucuronidase activity, with activation occurring preferentially in necrotic tumour cores. This principle has been extended to bioconjugates and ADC-like constructs, in which glycosidase-cleavable linkers release the payload only after enzymatic removal of the sugar and subsequent self-immolation of a spacer. Glycosidase-triggered release is particularly attractive for targeting necrosis-rich tumours, where extracellular enzyme levels are highest [16,46,47,48].

However, glycosidases are not tumour exclusive. They can be elevated in inflammatory lesions, sites of tissue remodelling, and certain normal organs with high lysosomal turnover. Their activity is highly heterogeneous within tumours and strongly dependent on the extent of necrosis and hypoxia. As a result, tumour selectivity arises from localized enzyme activity and microenvironmental context, rather than expression alone. These considerations both highlight the potential and the limitations of glycosidase-response linkers as tumour-selective activation strategies.

## 4. Design Principles for Enzyme-Cleavable Linkers

Design principles for enzyme-cleavable linkers in ADCs revolve around achieving a high differential between stability in circulation and efficient and selective cleavage in tumour or lysosomal compartments, while preserving favourable physicochemical properties and a robust therapeutic index. Enzyme responsiveness typically comprises three modular elements: a recognition motif, usually a peptide or glycosidic unit, that is selectively cleaved by a tumour-associated enzyme, a self-immolative spacer, most commonly para-aminobenzyl carbamate (PABC), that relays the cleavage event to release the free drug, and a flanking structural feature that tunes hydrophobicity, steric, and overall stability [6,18,23,63]. Here, the self-immolative spacers represent the dominant clinical strategy for these linkers, and their optimization has been the focus of extensive structure-activity relationship (SAR) work over the past decade [63].

### 4.1. Peptide Linker Optimization

Dipeptide linkers of the Val-Cit type, usually coupled with a PABC spacer, are the most commonly enzyme-cleavable motifs and are incorporated in several approved ADCs, including Brentuximab vedotin, Polatuzumab vedotin, and Enfortumab vedotin. In these constructs, cathepsin B and other lysosomal cysteine proteases cleave the amide bond between the citrulline residue in the first position (P1) and the PABC unit (P1’), starting self-immolation of the spacer and release of the attached payload. Early studies revealed that these constructs strike a better balance between plasma stability and lysosomal cleavability, but they can also reveal susceptibility to species-specific enzymes, making preclinical evaluation difficult.

To address these limitations, linker design has expanded beyond simple dipeptides to tri- and tetrapeptide motifs, and modified dipeptides with altered side chains, to fine-tune cathepsin specificity and stability [16,17,32]. Systematic SAR studies have shown that modifying amino acid identity in the P1-P4 positions can significantly alter the rate and specificity of cleavage by lysosomal proteases. For instance, replacing valine at P2 with bulkier or conformationally constrained residues, or introducing β-branched amino acids at P3/P4, can modulate how well the peptide fits cathepsin S against cathepsin B, simultaneously reducing recognition by off-target proteases and esterases [16,18,23,32]. A prominent example is the GGFG tetrapeptide linker used in Trastuzumab-deruxtecan, which is characterized by high plasma stability and efficient cleavage by lysosomal cathepsins, enabling a DAR of 8 without compromising PKs.

These variations have also been used to engineer greater tumour vs. normal tissue selectivity by matching linker motifs to the substrate preferences of proteases enriched in specific tumours [23,24,64]. Substrate profiling and positional scanning libraries identified peptide sequences preferentially cleaved by tumour-associated cathepsins and lysosomal proteases, allowing design of linkers that are processed faster in tumour environments than in those of normal cells [24,65,66].

Critically, peptide linkers need to be optimized and aligned with the payload and overall ADC architecture: heavily hydrophobic payloads require more hydrophilic or longer linkers to avoid aggregation, whereas less lipophilic drugs can tolerate shorter linkers without compromising the overall architecture [6,18,23].

### 4.2. Self-Immolative Spacers and Payload Release

Self-immolative spacers represent a key feature for many enzyme-cleavable ADC linkers, as they decouple the initial enzymatic cleavage from the final release of the active payload, enabling the modular combination of different recognition motifs with diverse drugs [6,16,67]. There are several types of spacers, from variants of the p-aminobenzyl (PAB) scaffold to cyclisation-based spacers and even peptidic and heterocyclic spacers, but the most common and widely used in approved ADCs is the PABC one [68,69,70,71].

Under normal conditions, the protease recognizes and cleaves the peptide bond upstream of the spacer, creating an aniline or related intermediate that, after undergoing a rearrangement to release the payload in its native form, is critical for preserving potency and predictable pharmacology. This two-step process allows the same peptide trigger and spacer architecture to be combined modularly with multiple payloads, as long as the last one can be attached through a self-immolative linkage [16,17,67].

The electronics and substitution patterns of the self-immolative spacers strongly influence the rate of drug release and reduce undesired side reactions, like premature hydrolysis or payload modification, during circulation and manufacturing. The use of more complex self-immolative systems to enable the conditional release of payload under tightly defined biochemical conditions has been explored [67,71].

Finally, to ensure predictable clinical behaviour, the design must consider both the enzymatic step and the subsequent spontaneous fragmentation of the spacer under physiological conditions [67,72].

### 4.3. Physicochemical Impact

The interplay between payload hydrophobicity, linker composition, and overall ADC architecture represents a critical determinant of aggregation, clearance, and off-target effects. Several clinically used payloads, such as auristatins and maytansinoids, are highly lipophilic, and the direct attachment through short or hydrophobic linkers can significantly increase the hydrophobic surface area of the antibody, leading to aggregation, non-specific binding, rapid hepatic clearance, and a higher uptake in normal tissues [6,18,32].

Linker design provides a powerful tool to try to mitigate these liabilities; however, they do not eliminate the risks of aggregation, rapid clearance, or hepatic uptake, as each ADC architecture must be seen individually. The incorporation of hydrophilic elements, such as short PEG chains, charged amino acids or polar spacers, into peptide linkers can reduce aggregation, enable higher DAR values and improve exposure without compromising enzyme access or cleavage [6,17,18,32]. For instance, introduction of PEG7 spacers or charged residues in a Val-Cit-PABC motif has been shown to increase solubility and support DARs of 6 to 8 while still maintaining acceptable PKs and efficacy [6,17]. In contrast, truncation of the linker or introduction of more hydrophobic residues often results in increased aggregation and faster clearance, resulting in a finely tuned balance between linker length, composition and developability [6,32].

Hydrophilicity is not only critical for manufacturability and PKs but also for maintaining ligand binding and target engagement. Overly hydrophobic linkers and high DARs can stimulate antibody–antigen interaction in non-target tissues or Fc-mediated uptake in liver and spleen, increasing off-target toxicity, whereas hydrophilic linkers can help preserve the native distribution of the unconjugated antibody [18,73]. Overall, hydrophilic linkers are specifically chosen to offset very hydrophobic topoisomerase- or tubulin-targeting payloads and thus maintain good PKs and safety profiles.

### 4.4. Steric Shielding and Unnatural Amino Acids

The gathered expertise on the peptides’ physicochemical properties leading to better proteolytic stability shall boost the design of stable peptides [25]. Beyond sequence optimization, steric and conformational shielding around the scissile bond provides an additional lever to enhance tumour selectivity and suppress off-target cleavage through non-intended proteases or serum enzymes [74]. Strategies include introducing bulky side chains, *D*-amino acids or non-canonical residues at non-critical positions adjacent to the cleavage site and using non-canonical residues that maintain recognition with specific tumour-associated proteases but are poorly tolerated by broader-specificity proteases in plasma or normal tissues [16,23,24]. These strategies can restrict access for broad-specificity proteases while preserving efficient processing by the target lysosomal cathepsin or tumour-associated protease [24].

Substitutions at P1’, the residue immediately after the cleavage site, or at flanking positions reduce off-target cleavage by serum proteases without significantly impairing cathepsin B or legumain activity [23,72]. Incorporating sterically demanding residues such as cyclohexylalanine (Cha) or norleucine (Nle) at P1’ reduces off-target cleavage by serum proteases without significantly impairing processing by cathepsin B or legumain. Similarly, the incorporation of *D*-amino acids at positions that are less critical for enzyme recognition has also been used to reduce cleavage by off-target proteases and prolong plasma stability [16,23,74].

Recent peptide-linker designs have started to use non-canonical amino acids and peptidomimetics to sculpt the local environment around the cleavage site so that only the intended tumour-associated protease can efficiently bind and process the linker [65,66]. These approaches take advantage of small changes in S1-S4 pocket topology between protease isoforms, using side-chain size, polarity and conformational constraints to enhance selectivity. This design usually results in linkers with markedly improved human plasma stability, reduced interspecies variability and preserved lysosomal processing, supporting their potential as next-generation clinical linkers [16,65,66,72].

### 4.5. Multi-Trigger Linkers

Emerging designs extend beyond single-enzyme trigger systems to incorporate multi-stimuli or “logic-based” architectures that integrate enzymatic recognition with other TME cues, like pH, redox state or reactive oxygen species. In these systems, drug release may require sequential or concurrent activation steps, like an initial enzymatic cleavage and a secondary trigger such as acidic pH, reducing conditions or reactive oxygen species, effectively implementation and/or logic to further reduce the probability of payload release in normal tissues [24,63,75,76,77].

Even though most of these multi-trigger systems have been developed in the context of polymeric or nanoparticle drug delivery systems rather than classical ADC, the underlying principles are directly transferable to linker design [75,76,78]. For example, an ADC linker can be engineered to be first cleaved by cathepsin B, exposing a second masked peptide segment that will then be processed through a different lysosomal enzyme, or have a protease cleavage generating a self-immolative spacer that only fragments under acidic pH, thereby ensuring that the payload will only be fully released in the lysosomal compartment [75,77,79]. Likewise, dual-trigger linkers may require both protease activity and high glutathione concentration to finish self-immolation, decreasing the probability of payload release in extracellular or poorly reducing environments [63].

Designing these logic-based systems requires careful consideration of trigger hierarchy, kinetics, spatial localization, knowledge about tumour protease and glycosidase profiling, pH mapping and redox state characterization, as well as leveraging site-specific conjugation and computationally guided linker design. As these tools evolve, the multi-trigger enzyme-responsive linkers acquire the potential to provide an additional layer of specificity above antigen expression and single-enzyme activation, allowing more precise and robust tumour-selective payload release in next-generation ADCs [24,77,80].

### 4.6. Computational Engineering

Computational engineering is becoming an important tool for generating tumour-selective linkers, complementing experimental profiling approaches. Researchers started to use computational engineering techniques since they reduce the experimental search time and enable an iterative “design-build-test-learn” cycle for enzyme-cleavable ADC linkers that is more systematic and tumour-adapted than traditional trial-and-error medicinal chemistry. Computational engineering methods are generally based on machine learning and optimization techniques.

Machine learning techniques have been highly considered to generate tumour-selective linkers. Rezaee K. provides a comprehensive review that explores how the automated design of linkers can be enhanced by leveraging advanced machine learning and deep learning techniques [81]. Other reviews about the recent progress in linkers’ design using these techniques were provided by Yan J. and Wan F. [82,83]. Data from N-terminomics, PICS-type specificity assays, and biochemical cleavage studies provide sets of known protease substrates which can be used to train machine-learning models that predict cleavage probability for candidate P1-P4 sequences in specific proteases [84,85,86].

Recent work on artificial intelligence (AI)-assisted design demonstrates that these models can propose novel peptide motifs and full linker scaffolds with improved predicted stability and enzyme selectivity, including constraints on hydrophobicity and length. Su A. integrates transfer learning from large-scale molecular datasets and reinforcement learning to iteratively refine molecular linkers’ properties [22]. In parallel, molecular docking and molecular dynamics simulations help rationalize how candidates fit into pockets of tumour-associated proteases, guiding focused modifications to enhance binding to the target protease while discarding off-target enzymes [22,33,86]. Different optimization techniques, namely metaheuristics, are considered.

Metaheuristics have been recognized as having great importance to help researchers in finding solutions for some unsolvable problems or to discover better solutions for those problems that were already solved. Calvet L. discusses how metaheuristics are being applied to solve different bioinformatics optimization problems, namely the molecular docking problem [87]. Muhaxhiri Z. considers a simulated annealing approach [86]. Masoudi-Sobhanzadeh introduced a novel Pareto front-based algorithm for protein-peptide docking [88]. Dao S. proposes a combination of Particle Swarm Optimization and Generalized Normal Distribution Optimization to predict antimicrobial peptide toxicity [89]. Metaheuristics are sometimes combined with machine learning techniques, as in Boone K., where a genetic algorithm is combined with machine learning strategies for designing antimicrobial peptides [90].

## 5. Strategies for Tumour Specificity

Tumour specificity in enzyme-cleavable linkers depends not only on choosing the right substrate but also requires matching the linker sequence and architecture with the specific protease activity landscape of a given tumour type and the precise type of cleavage. There are three key ways to achieve this specificity: (i) deep protease profiling, to define tumour-based substrate preferences, (ii) using tri- and tetrapeptides instead of simple dipeptides, to better exploit these preferences, and (iii) control of cleavage mode (exo or endo) [16,23,33,91].

### 5.1. Deep Profiling (TAILS and Related Methods)

Deep profiling strategies aim to move from guesswork to quantitative maps of protease specificity and activity in specific tissues and are subsequently used to design tumour-biased linker sequences [84,85].

N-terminomic approaches, like TAILS, can capture and enrich newly formed N-termini in complex samples, allowing mass-spectrometry (MS) identification of endogenous protease cleavage sites in their native context [92,93,94]. These kinds of strategies have been applied to diverse systems, including human tissues and cell models, to profile substrates and cleavage motifs of MMP and other proteases under physiological or disease conditions [84,92]. In both cancer and inflammatory disease, TAILS has revealed distinct N-terminal and substrate signatures that differ between disease and healthy tissues, demonstrating that disease-associated proteases can reshape the pattern of proteolysis in a way that can be mined for selective substrates [93,94].

These strategies complement PICS and related global specificity assays, which use proteome-derived peptide libraries to define the P4-P4’ preferences of a protease’s active site [65,85]. Combining PICS-style specific data with TAILS-derived in-tissue cleavage sites enables the identification of motifs that are both intrinsically preferred by a specific protease and demonstrably cleaved only in a disease context [33,65,94]. With respect to ADC linker design, this yields candidate P1-P4 sequences that should be cleaved efficiently in tumour lysosomes or the TME but less in normal tissues, increasing the likelihood of a tumour-biased activation [16,33].

### 5.2. Tri-/Tetrapeptides Versus Dipeptides

Most approved ADCs with enzyme-cleavable linkers still rely on short dipeptide motifs, like Val-Cit coupled with a PABC spacer, which are recognized by lysosomal cathepsins. These linkers provide a good baseline performance; however, they present relatively broad sensitivity to multiple cathepsins and, sometimes, to non-target enzymes, which can reduce stability and make translation difficult [16,32].

Recently, researchers have started looking at tri- and tetrapeptides that provide a much richer design space for tumour specificity. Extending the peptide sequence allows engagement of the full S1-S4 subsite architecture of the protease and incorporation of specific residues to exploit subtle differences in pocket topology between protease isoforms or between tumour and normal tissues [3,23,33,95]. Some ADCs have already started using these types of linkers, like Trastuzumab deruxtecan, which uses the tetrapeptide GGFG, and it contributed to the ADC’s strong bystander effect in solid tumours [23]. Other types of tetrapeptide motifs, like EVCit or EEVCit, presented enhanced stability and tuned specificity relative to the classical Val-Cit dipeptide [23,33].

Tri- and tetrapeptide linkers also allow incorporation of tumour-biased motifs identified by substrate profiling. For instance, sequences enriched in solid tumours by TAILS or MSP-MS can be placed at P3-P4, while maintaining a cathepsin-preferred P1 residue, to create linkers that are cleaved more quickly in tumour lysosomes than in normal cells [33,65,96]. Having linkers that go beyond simple dipeptides is critical to exploit the full discriminatory potential of cathepsin B against other cathepsins and off-target proteases.

Another important aspect is species selectivity, and here some engineered tri-/tetrapeptides, usually including acidic residues or non-canonical side chains, are less susceptible to rodent esterases while still retaining human cathepsin B or L cleavage, improving the predictive value of preclinical models [16,33]. Exo-linker platforms that reposition extended tetrapeptides to more solvent-exposed positions further enhance this effect by reducing unwanted interactions with off-target enzymes [91,97]. Overall, moving from di- to tri-/tetrapeptides enables more nuanced control over enzyme selectivity, plasma stability, and tumour versus normal cleavage profiles than is possible with minimal dipeptide motifs.

### 5.3. Exo- Versus Endo-Cleavage

Recently, advances in linker engineering have expanded the focus of ADC design beyond linker stability alone to encompass the precise topology of protease-mediated cleavage, being processed by proteases that cleave on the internal bond between P1 and P1’, whereas new “exo-cleavable” linkers reposition the scissile bond and peptide segment relative to the self-immolative spacer [16,91].

In classical Val-Cit-PABC linkers, cathepsins cleave the peptide bond between Cit and PABC, forming an aniline intermediate that self-immolates to release the payload. This design works properly but can suffer from some liabilities: the cleavage site is relatively accessible to multiple cathepsins, and the local environment around the scissile bond is constrained by the need to keep both enzyme recognition and efficient self-immolation [16,32,33].

By contrast, exo-cleavable linkers present an alternative structure where the cleavable peptide is repositioned at an “exo” location relative to the PABC motif. This structure aims to better expose the peptide to lysosomal cathepsins while shielding it from plasma enzymes and reducing steric constraints on the self-immolative step [91,97]. These types of linkers have been reported to have increased plasma stability and reduced neutrophil elastase-mediated cleavage, while still retaining efficient intracellular processing and payload release. These linkers also appear to maintain or even improve antitumour efficacy, reduce off-target activation, and support higher DARs with hydrophobic payloads, by mitigating local hydrophobic clustering [91,98,99].

The distinction between “exo” and “endo” cleavage is not only geometric but has specificity implications. By repositioning the cleavage motif away from the tightly packed PABC-payload junction and altering its presentation, exo-linkers may alter which proteases can physically access and efficiently process the peptide [97,99]. This creates another adaptable parameter for tumour specificity: exo-positioned tetrapeptides can be optimized to be ideal substrates for specific enzymes in tumour cells, but poor substrates for extracellular proteases or plasma enzymes [97]. Moreover, due to exo-linkers being able to exploit longer, more hydrophilic sequences without compromising self-immolation, they are well suited to incorporate tumour-biased motifs defined by deep profiling while maintaining favourable ADC development [91,97,99].

Taken together, strategies for tumour specificity in enzyme-cleavable linkers now act on multiple levels, and when they converge, they offer a path toward truly tumour-adaptative linkers that go beyond generic Val-Cit motifs to exploit the specific degradome of each tumour type.

## 6. Challenges and Limitations

The transition from conventional linkers to increasingly sophisticated linkers requires more than just optimizing cleavage kinetics in a buffer. Clinically viable linker platforms must also demonstrate robust manufacturability, reproducible behaviour across species, favourable safety profiles and resistance to premature activation in vivo. These challenges are particularly pronounced for enzyme-cleavable linkers because of their dependence on biological triggers, including proteases, pH gradients or redox gradients, which differ across different tissues, tumour types and species [16,24,32].

### 6.1. Off-Target Cleavage

The main challenge is the off-target activation of cleavable linkers in healthy tissues or plasma, which can lead to premature payload release and systemic toxicity. Classical linkers, such as Val-Cit, were initially designed to be stable in circulation and specifically processed by cathepsin B or related proteases in lysosome [32,100]. However, subsequent studies demonstrated that these linkers might be susceptible to cleavage by other enzymes, like Ces1c in rodents, resulting in marked instability in rodent plasma. Susceptibility to Ces1c is a limitation of certain murine models and must not be generalized to other models [100,101]. Such species-specific enzymatic liabilities complicate the interpretation of preclinical PK, efficacy and toxicology studies, as linker stability observed in animal models may not accurately predict clinical behaviour in humans [102]. Thus, the information gathered with specific animal models should be presented separately and interpreted according to their specificities [103].

Several engineering strategies, like adding polar or acidic residues N-terminal or rearranging the linker structure to exo-cleavable linkers, were developed to try to mitigate these differences, making the linkers more stable in Ces1c-containing plasma while maintaining cathepsin-mediated activation in tumour cells [91,97,100]. These changes need to be guided by comparative stability tests in both human and animal extracts, to avoid over- or under-estimating clinical stability based on animal data alone.

Nevertheless, off-target cleavage does not occur only within species; it can also occur within species through extracellular proteases in the TME or in inflamed normal tissues. Some tumour-associated proteases can activate prodrugs and conjugates in the extracellular space, which can be beneficial when bystander killing is desired, but can cause on-target off-tumour and off-target toxicities when these enzymes are active in normal tissues [24,104]. Deep profiling of protease activity across tumour and normal tissues has revealed that some tumour-associated proteases are also active in normal organs, highlighting the need to match linker motifs to truly tumour-biased enzyme activity profiles [66,84,96,105].

This way, in studies about linker stability and cleavage assays, it is recommended to use multi-species panels combined with protease-profiling datasets to de-risk species-dependent off-target activation.

### 6.2. Multiple-Drug Antibody–Drug Conjugates

Tumour heterogeneity is a major driver of therapeutic failure, as different cellular subpopulations within the same tumour often display variable antigen expression, distinct mechanisms of resistance and divergent sensitivities to cytotoxic agents [14,106,107]. As a result, single payload ADCs may eliminate only one part of the tumour, allowing resistant clones to persist and ultimately cause relapse [108].

Multi-payload ADCs address this limitation by delivering mechanistically complementary drugs within a single targeted molecule, increasing the likelihood of killing heterogeneous cell populations and reducing the probability that cancer cells develop simultaneous resistance to both agents [109]. Recent studies have shown that dual-payload ADCs, combining MMAE and MMAF [110], achieve superior efficacy and survival benefit in models of intratumor heterogeneity and drug resistance, when compared to single-payload ADCs [111].

Although these formats introduce greater synthetic complexity and may require careful control of hydrophobicity and DAR, they offer a promising strategy to overcome tumour diversity and enhance the durability of ADC response [112]. Additionally, the methods for generating such constructs are being extensively optimized since they are numerous and complex owing to the plethora of reactive functional groups that make up the surface of a mAb. They can range from branched linker installation to the incorporation of unnatural amino acids [111].

### 6.3. Synthetic Complexity, Scalability, and Manufacturing

Complex peptide linkers and multi-trigger architectures also present significant chemistry, manufacturing, and controls challenges. Longer tri- and tetrapeptide linkers, especially those incorporating non-canonical amino acids or multiple functional modules, increase synthetic complexity and may require multi-step solid-phase synthesis followed by sophisticated purification to achieve GMP-grade quality [18,23]. Each additional step introduces potential impurities and errors that need to be controlled and characterized, raising analytical burden and cost [113].

From a regulatory perspective, ADCs are viewed as complex biological-chemical combination products and are expected to have a full characterization of both the linker–payload intermediate and the final conjugate. Critical quality characteristics include DAR and its distribution, the identity and abundance of positional isomers, levels of free payload and linker, and payload bond under storage and physiological conditions [19,114]. Multi-trigger links that contain multiple bonds increase the challenge for consistent manufacture and long-term stability, since each functional group must be shown to behave predictably within the target shelf-life and under stress conditions [113,114,115].

A critical aspect in ADC production is the consistency of the process, because variability in linker synthesis or conjugation can shift DAR distributions or create new degradation pathways that alter exposure and toxicity. The linkers are supposed to present comparable DAR profiles, impurity patterns, and linker–payload integrity, both in clinical and commercial batches, and sometimes, for that to happen, it is needed to optimize the formulation to maintain product quality and manufacturability [113,114,115].

Finally, it is important to consider the supply chain and cost. The production of custom linkers increases lead times and cost of goods, which can be particularly challenging for indications requiring high doses or chronic administration. As a result, industrial ADC programs tend to choose motifs that can be manufactured reproducibly at scale and integrated into platform conjugation processes [113,114,115,116,117,118,119,120].

### 6.4. Safety, Immunogenicity and Resistance

Linker behaviour is intrinsically related to ADC safety profiles, especially for cleavable linkers that generate membrane-permeable payloads capable of bystander effects [4]. Cleavable linkers that are too labile in plasma or normal tissues are more prone to increase systemic free payload levels, which in turn will accumulate in sensitive organs, exacerbating toxicity [104]. Contrarily, non-cleavable linkers generally present more favourable tolerability, but at the cost of reduced bystander activity and reduced efficacy in heterogenous solid tumours [102].

Although advantageous in some cases, bystander effects can be a double-edged sword. Lipophilic payloads released by cleavable linkers can diffuse from antigen-positive tumour cells into neighbouring antigen-negative cells, being beneficial for treating antigen-heterogeneous tumours, but it is also at risk of damaging normal cells in the tumour proximity [104]. Linker properties need to be tuned with payload, through linker modifications that adjust payload polarity or charge upon release, to balance intra-tumoral coverage with minimization of collateral damage in normal tissues [4,6,20].

Although clinical experience suggests that most modern ADCs present a low immunogenic profile when based on humanized or fully human antibodies, it is necessary to take into account immunogenicity because linkers and linker–payload constructs can create novel epitopes or alter antigen processing and presentation [4,115]. This way, monitoring the formation of anti-drug antibodies and assessing their potential impact on PK, efficacy, and safety over time is essential [114,115].

Finally, tumour resistance mechanisms can appear at multiple steps, such as downregulation or mutation of the target antigen, reduced internalization, altered endocytic routing, impaired lysosomal fusion or acidification, changes in protease expression, and upregulation of drug efflux pumps [54,121,122]. Therefore, linker design needs to anticipate these adaptive responses by introducing some redundancy, like dual protease sensitivity or microenvironment-responsive elements, to sustain long-term therapeutic efficacy. Pairing ADCs with efflux inhibitors or lysosome modulators further mitigates resistance and enhances clinical durability across diverse tumour types [123,124].

## 7. Conclusions

Over the past two decades, intense research in the development of ADCs has been performed, which has made clear that the linker is not just a mere connection between the payload and the antibody, but the central control element of the entire conjugate. What started with just simple hydrazone and disulfide linkers has now matured into a sophisticated design space of enzyme-cleavable peptides, self-immolative spacers, hydrophilically balanced scaffolds, and multi-trigger architectures that together control when, where and how a payload is released. As clinical experience with ADCs has improved, the main lesson is consistent: meaningful improvements in efficacy and safety rarely come from payload potency alone; it comes from co-optimization of target biology, conjugation strategies, and, critically, linker chemistry.

Enzyme-cleavable linkers are the centre of this progress. Owing to their ability to harness lysosomal proteases, cathepsins, MMPs, and glycosidases that are overexpressed or mislocalized in tumours, these linkers encode a biochemical layer of selectivity that complements antigen targeting. Modern peptide linkers go beyond generic dipeptide motifs to tri- and tetrapeptides, allowing them to be tailored to the S1-S4 preferences of specific proteases, improving plasma stability and influencing cleavage toward tumour lysosomes or microenvironments. Self-immolative spacers decouple enzymatic recognition from payload release, promoting modular attachment of diverse drugs whilst preserving tight control over fragmentation and catabolite structure. Simultaneously, a better understanding of how linker hydrophobicity and charge govern aggregation, DAR distribution, and clearance has driven the adoption of more hydrophilic linkers, enabling them to support higher DARs for hydrophobic payloads without compromising developability.

The main translational obstacles are now better defined, enabling new developments in linker design. Species-dependent off-target cleavage has prompted the development of Glu-Val-Cit and exo-cleavable structures, which have improved cross-species stability and highlighted the need to have systematic multi-species protease and stability panels. Manufacturing control and regulatory expectations around ADCs as complex combination products have forced the linkers to be not only pharmacologically right and elegant but also synthetically robust and analytically controllable, with consistent DAR, linker–payload integrity, and impurity profiles across clinical and commercial lots. Through safety analyses, it was discovered how linker choice and payload permeability determine the extent and radius of bystander effects, and thus the balance between killing antigen-heterogeneous tumours and sparing normal tissues. Lastly, the tumour resistance mechanisms, like modified internalization, lysosomal trafficking and protease expression, further highlight the need to consider cellular processing pathways when linkers are designed.

Forward-looking, linker design is becoming increasingly tumour-adaptive and data-driven, for which it needs deep profiling of tumour degradomes and glycosidase landscapes, combined with computational models trained on substrate libraries and proteomic datasets, to enable more rational selection of P1-P4 motifs and linker architectures with desired cleavage probabilities in specific enzymes and tissues. New exo-cleavable and multi-trigger linkers show how spatial repositioning and logical integration of enzymatic, pH and redox triggers can promote linker stability and selectivity beyond what single-trigger systems can achieve. When all of these tools and considerations are taken into account and integrated with site-specific conjugation, bispecific antibodies and dual-payload conjugates, enzyme-cleavable tumour-selective linkers are prompted to transform ADCs from broadly targeted biological deliveries into programmable precision devices capable of delivering distinct payloads with unprecedented biochemical and spatial control. Nevertheless, most of these strategies remain primarily preclinical, which means that they still need clinical validation before major claims can be made on their behalf.

## Figures and Tables

**Table 1:** Characteristics of approved ADCs.

Common Name	Trade Name	Target	mAb	Linker	Linker Type	Linking Technology	Payload	Payload Type	DAR	Approved Indications	Approval Date	Approval Institution
Brentuximab vedotin	Adcetris	CD30	IgG1	Mc-Val-Cit-PBAC	Cleavable	Cysteine	MMAE	Microtubule inhibitor	4	R/R HL, sALCL, CTCL, MF, PTCL	2011	FDA/EMA
Polatuzumab vedotin	Polivy	CD79b	IgG1	Mc-Val-Cit-PBAC	Cleavable	Cysteine	MMAE	Microtubule inhibitor	3.5	R/R DLBCL	2019	FDA
Enfortumab vedotin	Padcev	Nectin4	IgG1	Mc-Val-Cit-PBAC	Cleavable	Cysteine	MMAE	Microtubule inhibitor	3.8	UC	2019	FDA
Disitamab vedotin	Aidixi	HER2	IgG1	Mc-Val-Cit-PBAC	Cleavable	Cysteine	MMAE	Microtubule inhibitor	4	HER2+ GC, HER2+ UC	2021	NMPA
Tisotumab vedotin	Tivdak	TF	IgG1	Mc-Val-Cit-PBAC	Cleavable	Cysteine	MMAE	Microtubule inhibitor	4	R/M CC	2021	FDA/EMA
Trastuzumab deruxtecan	Enhertu	HER2	IgG1	MC-Gly-Gly-Phe-Gly-AM	Cleavable	Cysteine	Dxd	TOP1 inhibitor	8	GC, GEJC, HER2+ BC, HER2+ GC, HER (low) BC, HER2 (mut) NSCL	2019	FDA
Datapotamab deruxtecan	Datroway	TROP2	IgG1	MC-Gly-Gly-Phe-Gly-AM	Cleavable	Cysteine	Dxd	TOP1 inhibitor	4	mBC	2025	FDA
Loncastuximab tesirine	Zynlonta	CD19	IgG1	Mal-PEG8-Val-Ala-PABC	Cleavable	Cysteine	SG3199	DNA cleavage	2.3	BCL, R/R DLBCL	2021	FDA/EMA
Gemtuzumab ozogamicin	Mylotarg	CD33	IgG4	AcBut	Cleavable	Lysine	Ozogamicin	DNA cleavage	2–3	R/R CD33 + AML	2000	FDA
Inotuzumab ozogamicin	Besponsa	CD22	IgG4	AcBut	Cleavable	Lysine	Ozogamicin	DNA cleavage	6	R/R ALL	2017	FDA/EMA
Sacituzumab govitecan	Trodelvy	TROP2	IgG1	CL2A	Cleavable	Cysteine	SN-38	TOP1 inhibitor	7.6	BC, TNBC, UC	2020	FDA
Sacituzumab tirumotecan	Jiatailai	TROP2	IgG1	Pytimidine-CL2A-carbonate	Cleavable	Cysteine	Belotecan-derivative	TOP1 inhibitor	7.4	EGFRm NSCLC	2024	NMPA
Mirvetuximab soravtansine	Elahere	FRα	IgG1	Sulfo-SPDB	Cleavable	Lysine	DM4	Microtubule inhibitor	3.5	PROC, PRFTC, PRPC	2022	FDA/EMA
Trastuzumab emtansine	Kadcyla	HER2	IgG1	SMCC	Non-cleavable	Lysine	DM1	Microtubule inhibitor	3.5	BC, HER2+ BC, mBC	2013	FDA/EMA
Belantamab mafodotin	Blenrep	BCMA	IgG1	Maleimido-caproyl	Non-cleavable	Cysteine	MMAF	Microtubule inhibitor	4	R/R MM	2020	FDA

ADCs Antibody–drug conjugates, ALL Acute lymphoblastic leukemia, AML Acute myeloid leukemia, BCL B-cell lymphoma, BCMA B cell maturation antigen, CTCL Cutaneous T-cell lymphoma, DAR Drug-antibody ratio, DLBCL Diffuse large B-cell lymphoma, DM1 emtansine, DM4 ravtansine, DXd Deruxtecan, EGFR Epidermal growth factor receptor, EMA European Medicines Agency, FDA Food and Drug Administration, FRα Folate receptor alpha, GC Gastric cancer, GEJC Gastro esophageal junction cancer, HCL Hairy cell leukemia, HER2 Human epidermal growth factor receptor 2, HER2 + HER2-positive, HER2 (low) low HER2 expression, HER2 (mut) activating HER2 mutations, HL Hodgkin lymphoma, HNSCC Head and neck squamous carcinoma, mBC metastatic breast cancer, mAb Monoclonal antibody, MM Multiple myeloma, MMAE Monomethyl auristatin E, MMAF Monomethyl auristatin F, MF Mycosis fungoides, Nectin-4 Nectin cell adhesion molecule-4, NMPA National Medical Products Administration, NSCLC Non-small cell lung cancer, PMDA Pharmaceuticals and Medical Devices Agency, PRPC Platinum-resistant peritoneal cancer, PRFTC Platinum-resistant fallopian tube cancer, PROC Platinum-resistant ovarian cancer, PTCL Peripheral T-cell lymphomas, R/M CC Recurrent or metastatic cervical cancer, R/R Relapsed or refractory, sALCL systemic anaplastic large cell lymphoma, SMCC Succinimidyl trans-4-(maleimidylmethyl) cyclohexane-1-carboxylate, SN-38 7-ethyl-10-hydroxycamptothecin, TF Tissue factor, TNBC Triple negative breast cancer, TROP2 Trophoblast cell surface antigen 2, UC Urothelial cancer.

**Table 2:** ADCs in Phase III clinical trials.

Common Name	Target	mAb	Linker	Linker Type	Payload	Payload Type	DAR	Representative Indications	Phase	NCT Number	Outcome
Zilovertamab vedotin	ROR1	IgG1	mc-Val-Cit-PABC	Cleavable	MMAE	Tubulin binder	4	DLBCL	II/III	NCT06717347 NCT05139017	Ongoing
BNY323/DB-1303	HER2	IgG1	mc-Gly-Gly-Phe-Gly	Cleavable	P1003	TOP1 inhibitor	8	HER2+ BC, mBC, EC	III	NCT06265428 NCT06018337 NCT06340568	Ongoing
TQB-2102	HER2	IgG1	N/A	N/A	N/A	TOP1 inhibitor	5.8	BC	III	NCT06561607	Ongoing
Trastuzumab duo-carmazine/SYD985	HER2	IgG1	mc-PEG2-Val-Cit-PABA-Cys	Cleavable	Seco-DUBA	DNA-damaging	2.8	mBC	III	NCT03262935	PFS, 7.0 vs. 4.9 m OS, 20.4 vs. 16.3 m ORR, 27.8% vs. 29.5%
ARX-788	HER2	IgG1	Hydroxylamine-PEG4	Non-cleavable	MMAF	Tubulin binder	1.9	HER2+ BC	II/III	NCT05426486	Ongoing
FS-1502	HER2	IgG1	Geranyl ketone pyrophosphate oxime ligation	Cleavable	MMAF	Tubulin binder	2	BC	III	NCT05755048	Ongoing
MRG-002	HER2	IgG1	mc-Val-Cit-PABC	Cleavable	MMAE	Tubulin binder	3.8	aBC, mBC, aCU, mCU	II/III	NCT04924699 NCT05754853	Ongoing
DP303c	HER2	IgG1	PEG2-Val-Cit-PABC	Cleavable	MMAE	Tubulin binder	2	HER2+ BC, HER2+ aBC	III	NCT06313086 NCT05901935	Ongoing
SHR-A1811	HER2	IgG1	mc-Gly-Gly-Phe-Gly	Cleavable	SHR9265	TOP1 inhibitor	5.7	HER2+ BC, OC, CRC, HER2+ r/mBC, NSCLC, HER2+ GC/GEJC	III	NCT05814354 NCT06828354 NCT06057610 NCT06199973 NCT06430437 NCT06126640 NCT05424835 NCT06123494	Ongoing
Patritumab deruxtecan	HER3	IgG1	mc-Gly-Gly-Phe-Gly	Cleavable	Dxd	TOP1 inhibitor	8	NSCLC	III	NCT05338970	Ongoing
MRG-003	EGFR	IgG1	mc-Val-Cit-PABC	Cleavable	MMAE	Tubulin binder	3.8	HNSCC	III	NCT05751512	Ongoing
Depatuxizumab mafodotin/ABT-414	EGFR	IgG1	Maleimidocaproxyl	Non-cleavable	MMAF	Tubulin binder	3.8	GBM, GSM	III	NCT02573324	OS, 18.7 vs. 18.9 m
FDA018	TROP2	IgG1	N/A	N/A	SN-38	TOP1 inhibitor	7.6	TNBC	III	NCT06519370	Ongoing
Tusamitamab ravtansine/SAR408701	CEACAM5	IgG1	SPDB	Clavable	DM4	Tubulin binder	3–4	NSCLC	III	NCT04154956	PFS, 75.39 vs. 45.85 m OS, 12.8 vs. 11.5 m ORR, 21.% vs. 24.1 m
Telisotuzumab vedotin	MET	IgG1	mc-Val-Cit-PABC	Cleavable	MMAE	Tubulin binder	3.1	NSCLC	III	NCT04928846	Ongoing
Telisotuzumab adizutecan/ABBV-400	MET	IgG1	N/A	N/A	Adizutecan	TOP1 inhibitor	/	SCLC, ESCC	III	NCT07525206 NCT07155187	Ongoing
Oportuzumab monatox/Vicinium	EpCAM	scFv	N/A	N/A	ETA-252–608	Tubulin binder	/	BCa	III	NCT02449239	CRR, 40% DOR, 9.4 m
Sigvitatug vedotin/SGN-B6 A	ITGB6	IgG1	mc-Val-Cit-PABC	Cleavable	MMAE	Tubulin binder	4	NSCLCL	III	NCT06758401 NCT06012435	Ongoing
Ifinatamab deruxtecan	CD276	IgG1	mc-Gly-Gly-Phe-Gly	Cleavable	Dxd	TOP1 inhibitor	6	SCLC, ESCC	III	NCT06203210 NCT06644781	Ongoing
Raludotatug deruxtecan	CDH6	IgG1	mc-Gly-Gly-Phe-Gly	Cleavable	Dxd	TOP1 inhibitor	8	Solid Cancer	II/III	NCT06161025	Ongoing
Luveltamab tazevibulin/STRO-002	FRα	IgG1	Val-Cit-PABA	Cleavable	SC209	Tubulin binder	4	OC, FTC, PC	II/III	NCT05870748	Ongoing
Rinatabart sesutecan	FRα	IgG1	Cys-11	Non-cleavable	Exatecan	TOP1 inhibitor	8	PROC	III	NCT06619236	Ongoing

aBC, advanced breast cancer; ADCs, antibody–drug conjugates; AF-HPA, auri-statin hydrophile-polymer; aUC, advanced urothelium cancer; BCa, bladder cancer; CC, cervical cancer; CDH6, cadherin 6; CEACAM5, CEA cell adhesion molecule 5; CRC, colorectal cancer; DAR, drug-to-antibody ratio; DLBCL, diffuse large B-cell lymphoma; DM4, ravtansine; DXd, deruxtecan; EC, endometrial cancer; EF-2, elongation factor 2; EGFR, epidermal growth factor receptor; EpCAM, epithelial cell adhesion molecule; ESCC, esophageal squamous cell carcinoma; FRα, folate receptor alpha; FTC, fallopian tube cancer; GBM, glioblastoma; GSM, gliosarcoma; GEJC, gastroesophageal junction cancer; HER2, human epidermal growth factor receptor 2; HER2 + aBC, HER2-positive advanced breast cancer; HER2 + GC/GEJC, HER2-positive gastric cancer or gastroesophageal junction adenocarcinoma; HER2 + BC, HER2-positive breast cancer; HER2 + r/mBC, HER2-positive recurrent or metastatic breast cancer; HER3, human epidermal growth factor receptor 3; HNSCC, head and neck squamous carcinoma; ITGB6, integrin alpha V beta 6; mAb, monoclonal antibody; mBC, metastatic breast cancer; mCRC, metastatic colorectal cancer; MET, mesenchymal–epithelial transition factor; MMAE, monomethyl auristatin E; MMAF, monomethyl auristatin F; mUC, metastatic urothelium cancer; NSCLC, non-small cell lung cancer; OC, ovarian cancer; PC, peritoneal cancer; PROC, platinum-resistant ovarian cancer; ROR1, receptor tyrosine kinase-like orphan receptor 1; SCLC, small cell lung cancer; seco-DUBA, seco-duobamycin hydroxybenzamide-azaindole; SPDB, N-succinimidyl 4-(2-pyridyldithio) butanoate; TNBC, triple negative breast cancer (http://clinicaltrials.gov).

## Data Availability

Data is available upon request.

## Abbreviations

The following abbreviations are used in this manuscript:

ADC	Antibody-drug conjugate
AI	Artificial intelligence
DAR	Drug-to-antibody ratio
DDS	Drug delivery system
ECM	Extracellular matrix
EMA	European Medicines Agency
EMT	Epithelial-to-mesenchymal transition
FDA	Food and Drug Administration
mAb	Monoclonal antibody
MS	Mass spectroscopy
MMP	Metalloproteinase
SAR	Structure-activity relationship
PAB	p-aminobenzyl
PABC	para-aminobenzyl carbamate
PK	Pharmacokinetic
TME	Tumour microenvironment
